# Re-sheathing failure with Navitor during transcatheter aortic valve implantation: a case report

**DOI:** 10.1093/ehjcr/ytaf015

**Published:** 2025-01-21

**Authors:** Hiroshi Onoda, Hiroshi Ueno, Yuki Hida, Teruhiko Imamura, Koichiro Kinugawa

**Affiliations:** The Second Department of Internal Medicine, University of Toyama, 2630 Sugitani, Toyama, Toyama 930-0194, Japan; The Second Department of Internal Medicine, University of Toyama, 2630 Sugitani, Toyama, Toyama 930-0194, Japan; The Second Department of Internal Medicine, University of Toyama, 2630 Sugitani, Toyama, Toyama 930-0194, Japan; The Second Department of Internal Medicine, University of Toyama, 2630 Sugitani, Toyama, Toyama 930-0194, Japan; The Second Department of Internal Medicine, University of Toyama, 2630 Sugitani, Toyama, Toyama 930-0194, Japan

**Keywords:** Transcatheter aortic valve implantation, Navitor, Re-sheathing failure, Case report

## Abstract

**Background:**

Self-expanding valves used in transcatheter aortic valve implantation (TAVI) are designed to allow recapture and repositioning, facilitating optimal placement and mitigating conduction disturbances and paravalvular leakage. Here, we present a rare case in which the Navitor (Abbott Structural Heart, Santa Clara, CA, USA) could not be recaptured.

**Case summary:**

An 81-year-old Japanese woman with very severe aortic stenosis and a massively calcified nodule at the non-coronary cusp (NCC) underwent TAVI with a 25 mm Navitor valve. During the initial deployment attempt, non-uniform expansion (NUE) was observed on the NCC side when the valve was 80% deployed. An attempt was made to recapture and reposition the valve, but the delivery system capsule failed to fully re-sheath the prosthesis, leaving approximately one-third of the valve outside the capsule and preventing complete recapture. The Navitor was promptly redeployed while still within the basal ring. Following redeployment, the NUE resolved, and the valve was successfully positioned 3 mm below the basal ring on the NCC side and 4 mm below the left coronary cusp. We hypothesized that interference between the capsule tip and the calcified nodule on the NCC leaflet inhibited the re-sheathing process.

**Conclusion:**

This report documents a rare complication involving the failure to recapture the Navitor valve. In cases with large calcified nodules on the leaflet, caution is essential during the re-sheathing process. We strongly recommend re-deploying the prosthesis rather than attempting to remove it from the basal ring to minimize procedural risks and ensure proper valve placement.

**Learning points**

- To understand the rare complication of inability to recapture the Navitor valve in cases with calcified nodules on the valve leaflets.
- To raise awareness of the necessary precautions and procedures to follow in the event of a failed re-sheathing.

## Introduction

Transcatheter aortic valve implantation (TAVI) is an established therapeutic intervention for the management of severe aortic stenosis.^1–4^ Precise valve placement is essential to ensure optimal valve function and to mitigate the risk of complications such as paravalvular leakage (PVL), conduction disturbance, and device embolism. There are two types of TAVI valve: balloon-expandable (BEV) and self-expandable (SEV). Balloon-expandable valves offer easier access to the coronary artery but have a higher risk of valve ring complications in cases of the left ventricular outflow tract (LVOT) and severe valve ring calcification. Self-expandable valves are advantageous in cases of severe calcification, with a lower risk of valve ring rupture, but may increase the risk of residual perivalvular leak. Selection of the optimal valve requires careful consideration of patient, anatomy, and device-specific factors, including long-term durability, conduction abnormalities, and the potential for future intervention.^5^

The Navitor (Abbott Structural Heart, Santa Clara, CA, USA) is a novel SEV system, designed with enhanced flexibility, an advanced PVL control mechanism featuring the NaviSeal cuff, and a maintained pressure gradient in an intra-annular design.^6^ A key advantage of the SEV is its re-capturability during deployment, enabling repeated attempts to achieve optimal positioning. In this report, we present a rare and complex case in which the valve could not be recaptured into the delivery system, posing a significant procedural challenge.

## Summary figure

**Figure:**
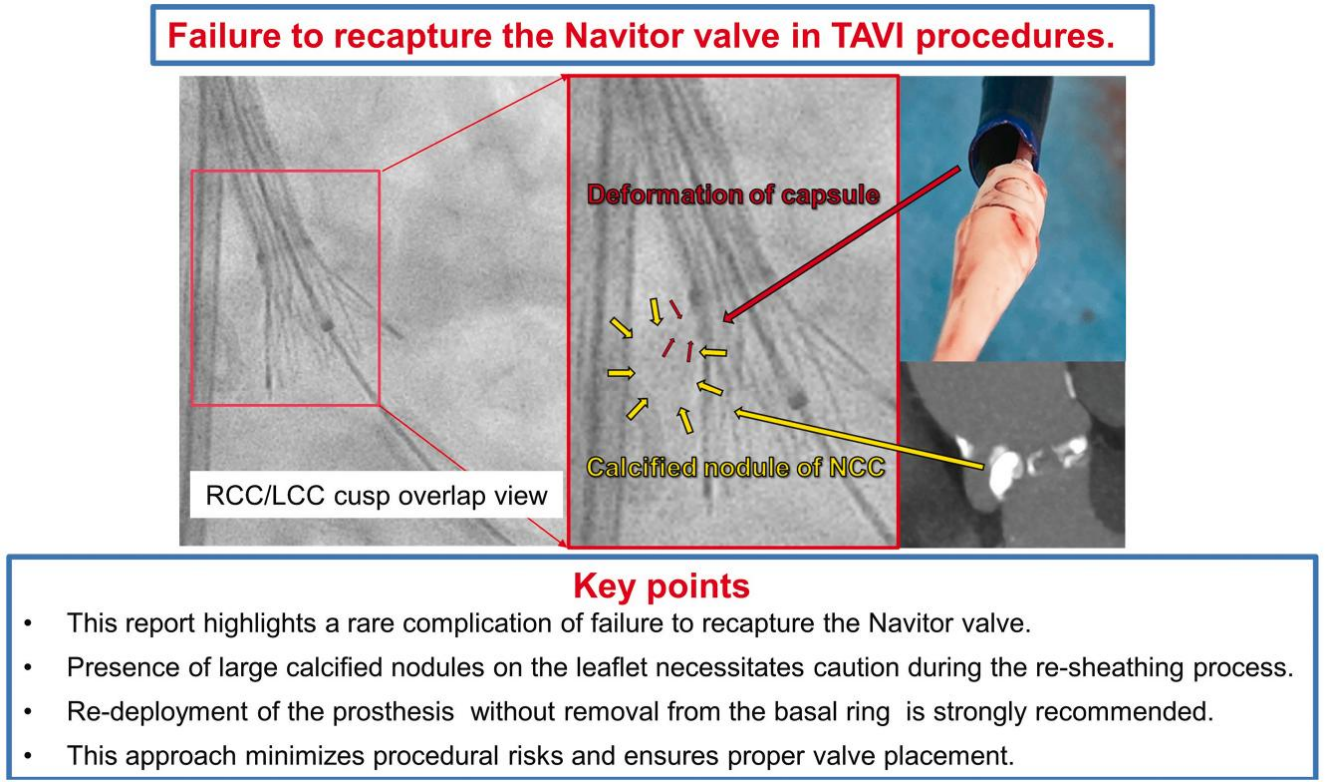


## Case presentation

### On referral

**Figure 1:**
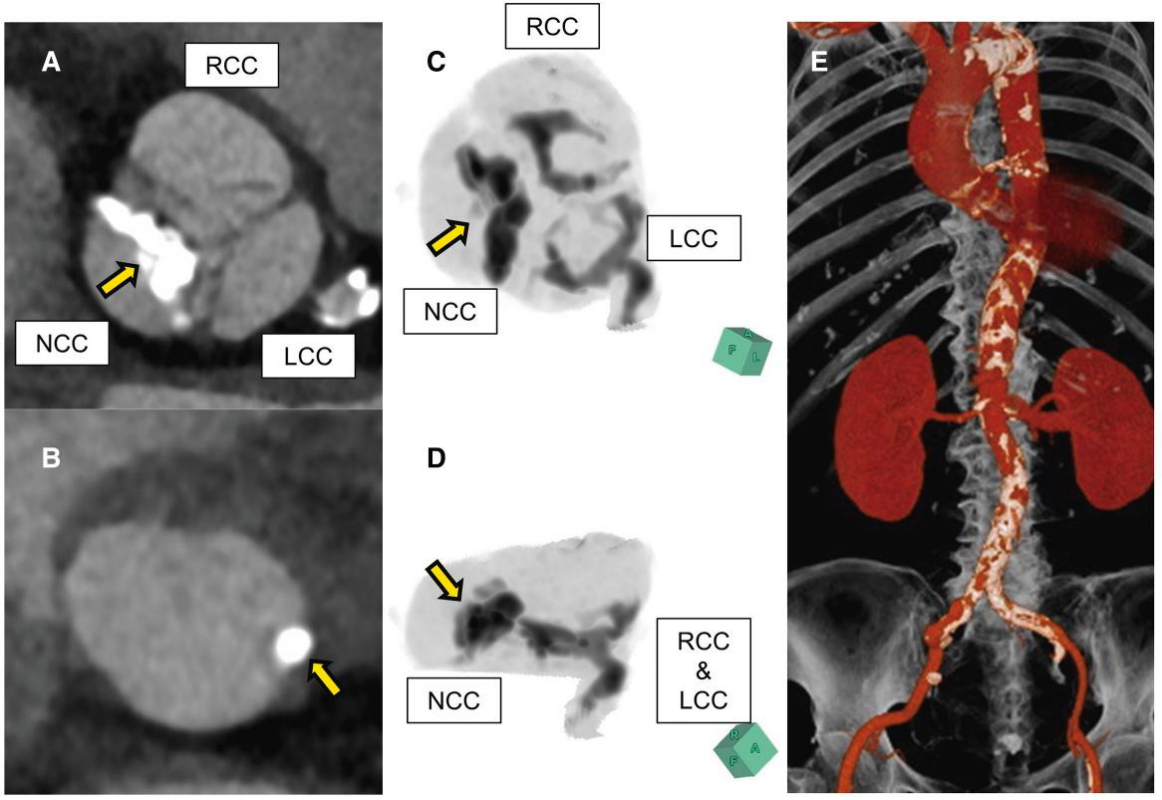
A pre-operative multislice computed tomography scan showed the following: Arrows indicate extensive calcification of the leaflet tips of the non-coronary cusps (*A*, *C*, and *D*). Arrows indicate continuous calcification of the left ventricular outflow tract (*B*). Computed tomography angiography revealed advanced calcification of the artery (*E*). NCC, non-coronary cusp; RCC, right coronary cusp; LCC, left coronary cusp.

An 81-year-old Japanese woman presented with exertional dyspnoea and was referred to our hospital for evaluation and treatment of very severe aortic stenosis. Her comorbidities included hypertension, type 2 diabetes, and stroke. The physical examination revealed a Levine grade 4/6 systolic ejection murmur at the second right sternal border, with no additional heart sounds detected. Bilateral lower extremity oedema was also present. Additionally, the N-terminal pro-brain natriuretic peptide level was elevated at 595 pg/mL. Transthoracic echocardiography showed a left ventricular ejection fraction of 70%, a peak aortic valve velocity of 51 m/s, a mean pressure gradient of 64 mmHg, and an aortic valve area of 0.3 cm^2^. No other valvular disease was identified. Coronary angiography showed no significant stenosis. Given her advanced age and frailty, TAVI was preferred to surgical intervention. A pre-operative computed tomography scan identified a massively calcified nodule at the non-coronary cusp (NCC) and the icicle-shaped calcification at the left coronary cusp (LCC) side of LVOT, prompting the selection of a SEV (*Figure 1A–D*).

The left common iliac artery (CIA) was occluded, with a minimum lumen diameter of the right CIA measuring 4.5 × 7.5 mm (*Figure 1E*). The aortic root angle was measured at 59 degrees. The Navitor valve was selected for its flexibility and trackability. The pre-operative computed tomography scan revealed a tricuspid valve with the following annular dimensions: a perimeter of 71.4 mm, an area of 384.7 mm^2^, a minimum diameter of 20.1 mm, a mean diameter of 22.6 mm, a maximum diameter of 25.1 mm, a circumference-based diameter of 22.7 mm, and an area-derived diameter of 22.1 mm. A 25 mm Navitor valve was selected for implantation.

### TAVI procedure

**Figure 2:**
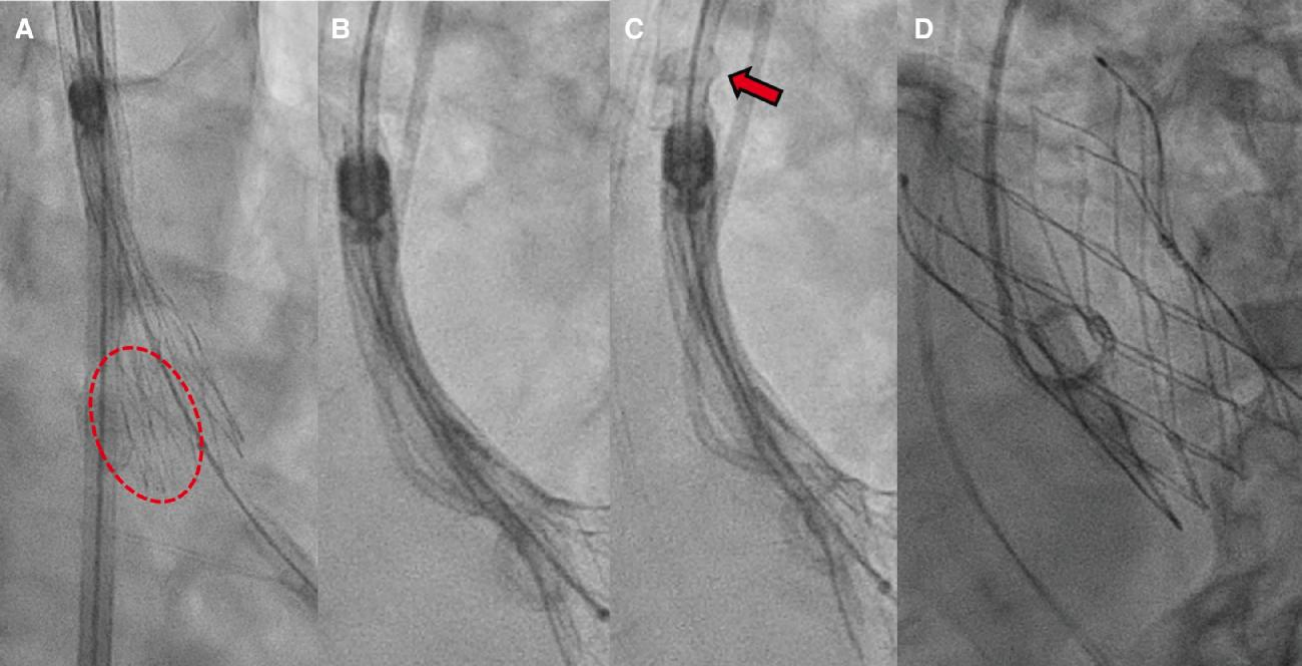
Fluoroscopic images of the first deployment, failed re-sheathing, and final deployment: non-uniform expansion in the dotted area during the first deployment (*A*), failed re-sheathing. The delivery system capsule has stopped re-sheathing (*B*). The arrows indicate capsule deformation (*C*). After prosthesis deployment (*D*).

After pre-dilation with an 18 × 40 mm Z-MED II balloon (NuMED, Canada) via the right femoral artery, we deployed the 25 mm Navitor valve under controlled pacing at 100 b.p.m. During deployment, non-uniform expansion (NUE) of the valve was identified (*Figure 2A*), prompting an attempt to recapture and reposition the prosthesis.

The re-sheathing procedure was initiated; however, approximately one-third of the Navitor valve remained outside the delivery capsule (*Figure 2B*). Despite the complete advancement of the re-sheathing ring handle, the delivery system capsule failed to re-sheath the valve, and the capsule exhibited deformation (*Figure 2C*). The Navitor was immediately redeployed while still within the basal ring (see Supplementary material online, *Video S1*).

Fortunately, the NUE resolved following redeployment, achieving optimal positioning with the prosthesis seated 3 mm below the basal ring on the NCC side and 4 mm below the LCC side (*Figure 2D*). The PVL caused by LVOT calcification was mild, and the patient exhibited no signs of atrioventricular block.

### After the procedure

**Figure 3:**
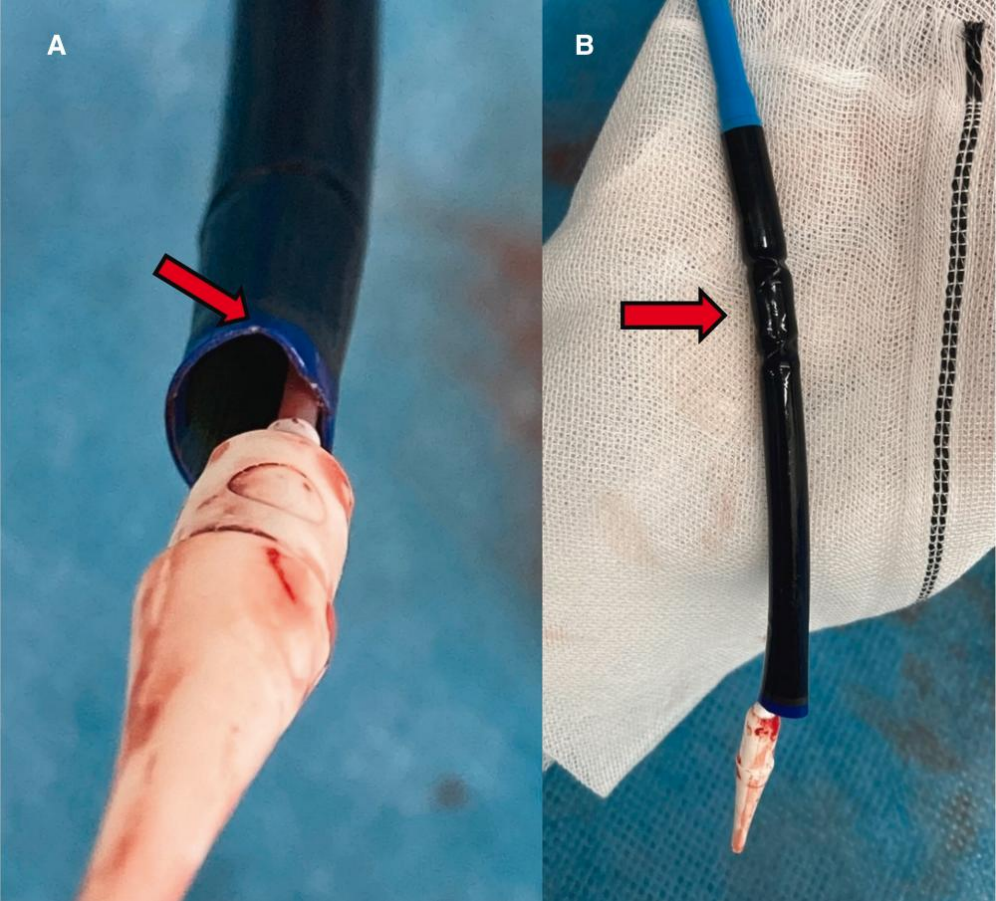
The Navitor delivery system after surgery. The tip of the capsule appears deformed, suggesting interference by the calcification at the site of the red arrow (*A*). The proximal part of the capsule is shortened, as indicated by the arrow (*B*).

Upon careful assessment of the removed device, it was noted that the proximal part of the capsule was shortened, with its tip dilated and deformed (*Figure 3*). We hypothesized that interference between the deformed capsule tip and the calcified nodules of the NCC leaflet prevented successful recapture of the valve.

She was discharged home independently on post-operative Day 7 after TAVI. Discharge echocardiography showed no PVL, a peak aortic valve velocity of 2.4 m/s, and a mean pressure gradient of 12 mmHg. N-terminal pro-brain natriuretic peptide levels decreased from 595 pg/mL to 75 pg/mL. Since then, the patient has continued to do well with no heart failure symptoms.

## Discussion

This case presents a patient with severe aortic stenosis with large calcified nodules on the NCC leaflets and left CIA occlusion, necessitating careful TAVI strategy consideration. The latter precluded the use of a contralateral femoral approach to provide extracorporeal membrane oxygenation support in the event of acute annular rupture, a known complication of BEV in patients with severe calcification. Given the severe calcification, SEV was selected to minimize the risk of annular rupture. Navitor was selected here because it is designed to reduce the risk of PVL, which is a concern in patients with severe calcification, especially with NaviSeal. However, a contingency plan for potential underexpansion of SEV, including balloon reinflation after deployment, was carefully considered.

The inability to re-sheath the Navitor is an exceptionally rare complication, with no documented cases in the literature, apart from a documented instance involving difficulties in re-sheathing a conventional self-expanding Evolut prosthetic valve (Medtronic, Minneapolis, MN, USA).^7^ The Navitor system is specifically designed to permit re-sheathing and repositioning prior to full deployment. In the present case, NUE occurred, necessitating re-sheathing. Non-uniform expansion typically arises when the radial force of the device is insufficient, resulting in asymmetric dilatation of the valve inflow and leading to potential valve migration. Adequate pre-dilation and full distal opening are generally recommended to prevent NUE.^8,9^ However, in this instance, concerns about annular rupture due to calcified nodules in the LVOT, acute aortic regurgitation, and injury to the Valsalva sinus following dilation of the heavily calcified NCC prompted the use of an 18 mm Z-Med II balloon, which was smaller than the annulus’s minimum diameter. Consequently, sufficient anterior dilation was not achieved, likely contributing to the re-sheathing difficulty.

In the present case, the rigidity of the delivery system further complicated the procedure, as the capsule tip encountered resistance from a calcified nodule on the NCC leaflet. Given these challenges, we opted not to withdraw the partially deployed Navitor from the aortic annulus and instead re-deployed it, successfully eliminating the NUE. This approach represents a key procedural insight.

It is also important to note that the deformation of the capsule may have further impeded the re-sheathing process. Even if the valve had been removed from the basal ring, the shortened and deformed capsule might not have fully retracted, leaving a gap between the capsule and the nose cone. This gap could obstruct the system’s passage through the aortic valve or complicate retrieval from the patient’s body.

Based on this experience, we strongly recommend re-deploying the Navitor prosthesis in cases where re-sheathing is unsuccessful, rather than attempting to remove it from the basal ring. This strategy minimizes procedural risks and ensures the valve remains correctly positioned within the annulus.

## Limitation

The hypothesis that calcified nodules interfered with the re-sheathing mechanism of the Navitor is derived from observations in a single case and has not been validated through additional clinical cases or experimental studies. Further investigation, including larger case series or controlled studies, is necessary to determine whether similar risks may arise in other patients undergoing TAVI with the Navitor valve.

## Conclusion

This report documents a rare complication involving the failure to recapture the Navitor valve. In cases with significant calcified nodules on the leaflet, careful attention is essential during the re-sheathing procedure to minimize risks and ensure successful valve deployment.

## Lead author biography


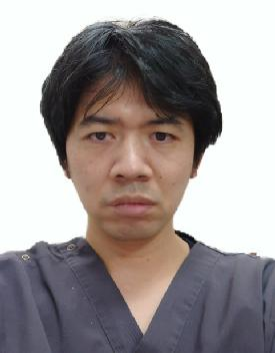


Dr Hiroshi Onoda obtained his MD and PhD degrees from the University of Toyama in Toyama, Japan. He has been working as a cardiologist specializing in ischaemic heart disease and structural heart disease at the Second Department of Internal Medicine, University of Toyama, since 2012.

## Supplementary Material

ytaf015_Supplementary_Data

## Data Availability

The data underlying this article are available in the article and in its online Supplementary material.

## Notes

**Consent:** The authors confirm that written consent for submission and publication of this case report, including images and associated text, has been obtained from the patient in line with COPE guidance.

**Funding:** None.
